# C-Type Lectin-Like Receptors: Head or Tail in Cell Death Immunity

**DOI:** 10.3389/fimmu.2020.00251

**Published:** 2020-02-18

**Authors:** Marion Drouin, Javier Saenz, Elise Chiffoleau

**Affiliations:** ^1^Université de Nantes, Inserm, Centre de Recherche en Transplantation et Immunologie, UMR 1064, ITUN, Nantes, France; ^2^OSE Immunotherapeutics, Nantes, France

**Keywords:** C-type lectin-like receptors, dead cells, sterile inflammation, tissue injury, cross-presentation

## Abstract

C-type lectin-like receptors (CLRs) represent a family of transmembrane pattern recognition receptors, expressed primarily by myeloid cells. They recognize not only pathogen moieties for host defense, but also modified self-antigens such as damage-associated molecular patterns released from dead cells. Upon ligation, CLR signaling leads to the production of inflammatory mediators to shape amplitude, duration and outcome of the immune response. Thus, following excessive injury, dysregulation of these receptors leads to the development of inflammatory diseases. Herein, we will focus on four CLRs of the “Dectin family,” shown to decode the immunogenicity of cell death. CLEC9A on dendritic cells links F-actin exposed by dying cells to favor cross-presentation of dead-cell associated antigens to CD8^+^ T cells. Nevertheless, CLEC9A exerts also feedback mechanisms to temper neutrophil recruitment and prevent additional tissue damage. MINCLE expressed by macrophages binds nuclear SAP130 released by necrotic cells to potentiate pro-inflammatory responses. However, the consequent inflammation can exacerbate pathogenesis of inflammatory diseases. Moreover, in a tumor microenvironment, MINCLE induces macrophage-induced immune suppression and cancer progression. Similarly, triggering of LOX-1 by oxidized LDL, amplifies pro-inflammatory response but promotes tumor immune escape and metastasis. Finally, CLEC12A that recognizes monosodium urate crystals formed during cell death, inhibits activating signals to prevent detrimental inflammation. Interestingly, CLEC12A also sustains type-I IFN response to finely tune immune responses in case of viral-induced collateral damage. Therefore, CLRs acting in concert as sensors of injury, could be used in a targeted way to treat numerous diseases such as allergies, obesity, tumors, and autoimmunity.

## Introduction

**Figure 1:**
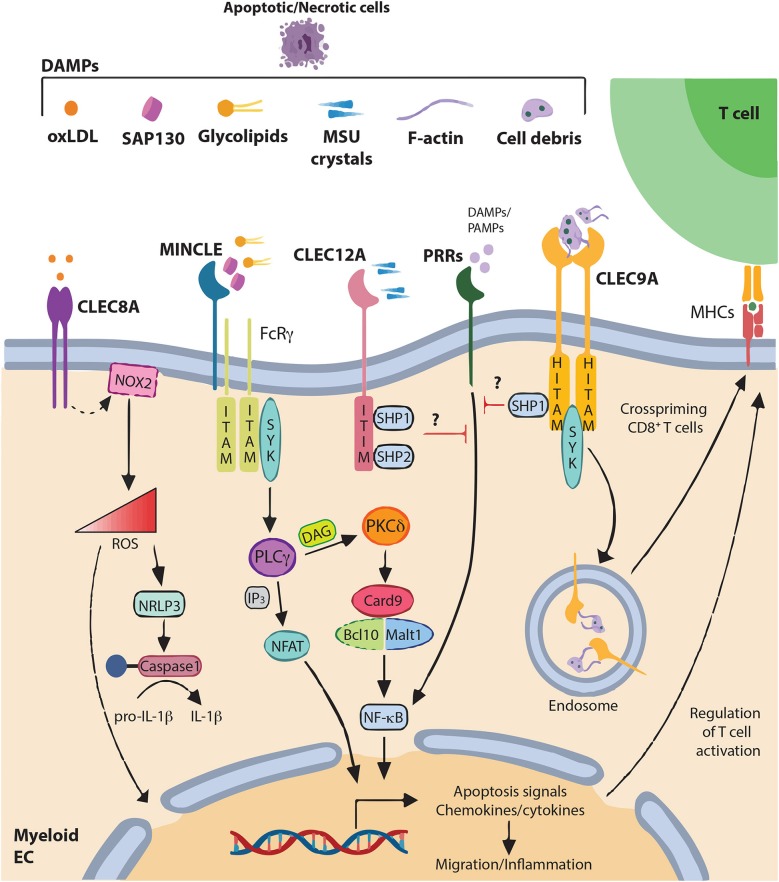
DAMPs recognition by C-type lectin-like receptors and signaling pathways C-type lectin-like receptors (CLRs) of the “Dectin family” recognize not only pathogen-associated molecular patterns (PAMPs), but also various self-derived ligands such as damage-associated molecular patterns (DAMPs). This recognition triggers activation of immune-receptor tyrosine-based activation motif (ITAM), leading to the recruitment and activation of SYK family kinases. Subsequent activation of the Card9–Bcl10–Malt1 complex through SYK induces NF-κB activation and gene transcription of various chemokines and cytokine (CLEC4E alias MINCLE). Alternatively, CLEC4E can also signal through PLCγ2 to induce the calcineurin/NFAT pathway. Alternatively, immune response can be regulated through increase of ROS and IL-1β production (CLEC8A) modifying gene expression and releasing ROS to the extracellular matrix. By contrast, activation of immune-receptor tyrosine-based inhibition motif (ITIM) induces the recruitment and activation of protein tyrosine phosphatases such as SHP-1 and SHP-2 and the dephosphorylation of activation motifs to inhibit cellular activation mediated by other pattern-recognition receptors (PRRs) (CLEC12A). CLEC9A, via Hemi-ITAM (HITAM) plays a key role in CD8^+^ T cell cross-priming. In addition, CLEC9A can activate SHP-1 to exert inhibitory feedback and restrain excessive immune response.

Cell death represents an important process occurring in the natural and physiologic contexts of embryonic development and tissue renewal, or in protection against factors such as disease or localized injury. In contrast to apoptosis, representing an orderly method of removing unwanted cells, necrosis is a more violent form of cell death that ultimately leads to the loss of integrity of the plasma membrane and the release of damage-associated molecular patterns (DAMPs) into the extracellular space (1–10). This induces an inflammatory response that subserves a number of biological functions exerting both positive and negative consequences. Inflammation will lead to rapid delivery of cellular and soluble defenses to the site of death in order to contain the injurious process and to help to clear debris and stimulate repair. However, release of anti-microbial molecules, such as reactive oxygen species by myeloid cells can further damage the tissue. Thus, inflammatory response can cause disease particularly in the case of dysregulated or excessive cell death (11). Molecules associated with dying/dead cells are detected by diverse receptors that via particular signaling, impact cell function and determine whether death is immunogenic or tolerogenic. Prominent among death sensors are the members of the C-type lectin receptor superfamily expressed mostly by myeloid cells (12). They constitute transmembrane and soluble receptors containing at least one carbohydrate recognition domain, in the broader sense, a C-type lectin-like domain (CTLD) (13–16). Via this domain, these receptors usually bind carbohydrates through a Ca2^+^ dependent conserved motif. However, some of them lack the Ca2^+^ binding site and are called C-Type Lectin-Like Receptors (CLRs). CLRs were shown to play an important role in both innate and adaptive immunity, particularly the ones from the “Dectin” family whose genes are localized in the telomeric region of the natural killer cluster (17, 18). Upon ligation, CLRs not only serve as antigen-uptake receptors for internalization and presentation to T cells, but also trigger multiple signaling pathways leading to NF-κB, type-I interferon (IFN) and/or inflammasome activation (17–20). CLRs are usually classified as activating or inhibitory receptors, based on their intracellular signaling motifs (21–23). They can have a classical immunoreceptor tyrosine-based activating motif (ITAM) constituted by YXXL tandem repeats in the intracellular tail or can interact with ITAM-containing adaptor proteins, such as Fc receptor γ (FcRγ) chain (21, 24). Other CLRs contain an hemi-ITAM motif composed of a single tyrosine within an YXXL motif (20, 25). Upon ligation, tyrosine(s) present in the ITAM or hemITAM motifs are phosphorylated, allowing the recruitment of SYK family kinases and the formation of the Card9/Bcl10/Malt1 complex (19, 21, 26–29). This leads to activation of NF-κB pathway and various cellular responses such as the production of reactive oxygen species (ROS) and the expression of diverse cytokines and chemokines to regulate both innate and adaptive immune responses (19, 28–33). In contrast, some CLRs contain an immunoreceptor tyrosine-based inhibitory motif (ITIM) that induces the recruitment of tyrosine phosphatases such as Src homology region 2 domain-containing phosphatase (SHP)−1 or −2, to negatively regulate the activity of activating signaling pathways (34–37). At last, some CLRs have neither ITAM nor ITIM domains and their signaling is either uncharacterized or utilizes alternative pathways such as via the serine/threonine kinase RAF-1 (17, 35, 38, 39). However, classification of CLRs as activating or inhibitory receptors is not as simple. A same CLR can according to the ligand (physical nature, affinity, avidity) or the environment, integrate distinct positive and negative signals, to shape immune response in complex scenarios (37). In addition, sensing of tissue damage by CLRs can complement detection of pathogens. This crosstalk in CLR recognition and signaling of both pathogen-associated molecular patterns (PAMPs) released following viral infections and DAMP from collateral injured cells may ensure microbial control while preserving integrity of the infected organs (40). Moreover, several studies have highlighted the fundamental role of these receptors during excessive cell death induced by sterile inflammation and their dysregulations were shown to lead to the development of inflammatory and auto-immune diseases (40–42). As shown herein in Table 1 and Figure 1, we will present a brief overview of the four CLRs of the dectin family largely described to decode the immunogenicity of cell death, thereby representing important medical therapeutic targets. This list is however not exhaustive. A recent study demonstrates that CLEC7A on DCs binds to annexins on apoptotic cells and induces by a selective SYK phosphorylation, the production of ROS to prevent auto-immune disease development (43).

**Table 1:** C-type lectin-like receptors sensing DAMPs.

	**Gene name**	**Expression**	**DAMP ligand/s**	**Functional effects**	**Associated diseases**
CLEC8A, OLR-1, LOX-1	*OLR1* (Hs) *Olr1* (Mm)	EC, mDC, moDC, B, MØ (Hs and Mm)	oxLDL, oxHDL, apoptotic bodies, phospha-tidylserine	↑ ROS, corpse uptake; ag capture and presentation	Promotes atherosclerosis, hypertension, diabetes, metabolic syndrome, coronary artery diseases and cancers
CLEC4E, MINCLE, CLECSF9	*CLEC4E* (Hs) *Clec4e* (Mm)	MØ, PMN (Hs and Mm)	SAP130, β-GlcCer, cholesterol sulfate and crystal	Necrotic cell uptake, ↑TNFα, IL-6, CXCL1, IL-1, MIP-1α/β, MIP-2	Promotes obesity, rheumatoid arthritis, allergic contact dermatitis, ischemic stroke, traumatic brain injury, hepatitis, sepsis and multiple sclerosis
CLEC12A, MICL, DCAL-2, CLL-1, CD371	*CLEC12A* (Hs) *Clec12a* (Mm)	CMP, GMP, MEP, MØ, Bs, Gr, Mo, DC (Hs and Mm)	Mono-sodium urate crystals	Necrotic cell ag cross-presentation, SYK inhibition, ↓CXCL1/10 and excessive neutrophil infiltration ↓ ROS, IL-8 ↑Type-I IFN	Reduces gout arthritis
CLEC9A, DNGR-1, CD370	*CLEC9A* (Hs) *Clec9a* (Mm)	CDPs; XCR1^+^ DC, pDC (Mm) BDCA3+ DC (Hs)	F-actin	Necrotic cell ag cross-presentation, ↓ MIP-2 and excessive neutrophil infiltration	Promotes atherosclerosis and pancreatitis

*CLRs reported to interact with DAMPs and discussed in this review. Hs, Homo sapiens; Mm, Mus musculus; CDPs, common DC progenitors; CMP, common myeloid progenitor; GMP, granulocyte myeloid progenitors; MEP, megakaryocyte-erythroid progenitors; Gr, Granulocytes; Bs, Basophils; MØ, Macrophages; Mo, Monocytes; B, B cells; EC, Endothelial cells; PMNs, polymorphonuclear leukocyte; moDCs, monocyte-derived DCs; mDC, myeloid DC; pDC, plasmacytoid DC; ag, antigen*.

## CLEC8A (LOX-1, OLR-1)

The lectin-like oxidized low-density lipoprotein receptor-1 (LOX-1) is a homodimer expressed by macrophages, DCs, B cells, endothelial cells, activated platelets and smooth muscle cells (17, 44–53). Its basal expression is relatively low but is dynamically upregulated by pro-inflammatory cytokines, stress, oxidized low-density lipoprotein (oxLDL), angiotensin II and endothelin (54, 55). LOX-1 is involved in numerous physiological functions and binds a broad spectrum of structurally distinct ligands including oxLDL (46), oxidized hypochlorite modified high-density lipoprotein (oxHDL) (56), phosphatidylserine (PS) (52), apoptotic bodies (57), advanced glycation end-products (AGEs) (58), bacteria (59), heat shock proteins 60 (Hsp60) (60), and platelets (61). LOX-1 has no known enzymatic or catalytic activity in its cytoplasmic tail and may require interaction with protein(s) for intracellular signaling. Following uptake of its ligand, LOX-1 induces pro-inflammatory signaling pathways leading to production of ROS, secretion of pro-inflammatory cytokines and induction of apoptosis signals (Table 1 and Figure 1). Thus, LOX-1 plays a pivotal role in the development of atherosclerosis, by inducing oxLDL uptake, lipidosis, foam cell generation and ultimately atheroma plaque formation (62, 63). This OxLDL/LOX-1 axis was also shown to play a role in cartilage degeneration during age-related osteoarthritis progression in the murine knee (64). Similarly, the triggering of LOX-1 by AGEs, contributes to diabetic complications such as atherosclerosis (65, 66). LOX-1 was also reported to play a key role in endothelial cell phagocytosis of apoptotic/aged cells due to the ability to recognize the exposure of PS on the surface of apoptotic cells (57). Interestingly, LOX-1 can be cleaved as a soluble form released in the circulation (67). This soluble form is overexpressed in patients with hypertension, diabetes, metabolic syndrome, and coronary artery diseases (58, 68), making soluble LOX-1 a non-invasive biomarker of disease. Importantly, numerous studies described the importance of LOX-1 in the progression of distinct types of cancer. Thus, a high expression of LOX-1 is correlated with a worse prognosis in patients suffering from gastric (69), colorectal (70), or prostate (71) cancer. Functionally, the triggering of LOX-1 by oxLDL was shown to induce TNF-α expression, tumor angiogenesis and tumor cell trans-endothelial migration and metastasis in prostate or breast cancers (71, 72). Interestingly, a high expression of LOX-1 was reported on polymorphonuclear myeloid-derived suppressor cells from peripheral blood and tumor of cancer patients (up to 5–50%), whereas its expression is almost undetectable on blood neutrophils from healthy donors (73). Therefore, expression of LOX-1 on these suppressive cells that is due to endoplasmic reticulum stress and lipid metabolism, may represent a specific therapeutic target in cancer. Besides, LOX-1 on DCs was shown to bind to Hsp to potentiate cross-presentation of chaperoned peptides (51) and apoptotic cells-coupled antigens (74) to cytotoxic CD8^+^ T cells. In addition, LOX-1 on B cells was shown to promote B cell differentiation into class-switched plasmablasts, their exit from germinal centers and their migration toward local mucosa and skin (48). Although the specific ligands remain to be characterized, this study demonstrates that triggering of LOX-1 could be applied for the design of new vaccines.

## CLEC4E (MINCLE, CLECSF9)

CLEC4E, more commonly known as MINCLE (Macrophage-inducible C-type lectin), associates with the FcRγ chain, an ITAM-containing adapter. In addition, it forms a functional heterodimer with Macrophage C-type Lectin (MCL). Through this complex, MINCLE is translocated to the plasma membrane and benefits the endocytic capacity of MCL to mediate an efficient phagocytosis. Moreover, through this complex, both receptors increase their affinity and specificity toward their ligands (75). MINCLE is expressed by antigen-presenting cells including macrophages, neutrophils, DCs and B cells (76). Its expression is induced by several inflammatory stimuli and stresses, such as lipopolysaccharide (LPS), tumor necrosis factor (TNF), IL-6 and saturated fatty acids (76–79) and was found over-expressed in numerous inflammatory diseases (77, 80–86). Interestingly, a polymorphism in this receptor has been linked to protection against rheumatoid arthritis in humans (87). MINCLE is largely described to recognize glycolipids from pathogens (88), but binds also ligands released by dead cells such as spliceosome-associated protein 130 (SAP130) (21, 89), β-glucosylceramide (β-GlcCer) (90), cholesterol sulfate and crystals (82, 91) (Table 1 and Figure 1). This triggers the recruitment of SYK and the activation of NF-κB, mitogen-activated protein kinase (MAPK), activator protein 1 (AP-1) or nuclear factor of activated T cells (NFAT) and downstream transcription of inflammatory genes (21, 88, 92–97). MINCLE induces the expression of several cytokines and chemokines such as TNF-α, IL-6, MIP-2, and CXCL1 (21, 28, 29). *In vivo*, MINCLE was shown to potentiate neutrophil infiltration following tissue damage induced by non-homeostatic cell death (21). Indeed, authors demonstrated that administration of anti-MINCLE blocking antibody following whole-body irradiation reduces MIP-2 production by thymic macrophages and consequently neutrophil infiltration. It remains to determine whether the recruitment of inflammatory cells induced by MINCLE is beneficial or exacerbates tissue damage. However, in an intriguing way, no such effect on neutrophilic inflammatory responses was observed in MINCLE-deficient mice following intra-peritoneal injection of necrotic cells or in response to liver cell necrosis induced by acetaminophen (98). One could speculate that this specific role of MINCLE on neutrophils may depend on the model or be due to some compensatory change with other dead-cell receptors in MINCLE-deficient mice. Alternatively, as neutrophils also express MINCLE, the use of antibodies may have exerted pleotropic effects by directly targeting or depleting neutrophils (98). Although MINCLE contributes to inflammation and immunity to contain the insult and initiate tissue repair, it can amplify collateral tissue damage and was therefore demonstrated to be implicated in numerous inflammatory diseases such as obesity, rheumatoid arthritis, allergic contact dermatitis, ischemic stroke, traumatic brain injury, hepatitis, sepsis, and multiple sclerosis (77, 80–86). Besides, MINCLE recognizes cholesterol crystals abundantly present in atherosclerotic plaques, triggering in macrophages the production of pro-inflammatory molecules (91). MINCLE was also shown to play a specific role on plasmacytoid DCs in skin allergies by recognizing cholesterol sulfate and inducing secretion of pro-inflammatory mediators such as IL-1α, IL-1β, MIP-1α, and MIP-1β (82). Furthermore, β-GlcCer whose accumulation leads to the systemic inflammation of Gaucher disease, was also characterized as a ligand for MINCLE, able to potentiate acquired immune response (90). Thus, MINCLE, via its high expression on M1-type macrophages and its ability to sense dead cells was also shown to be an important mediator of additional inflammatory diseases such as obesity and acute kidney injury (77, 78, 99, 100). In addition, MINCLE, by initiating inflammation, participates in the pathogenesis of cerebral ischemic stroke (83) and neuropathic pain by sensing damaged nerves (101). In cancer, the recruitment of macrophages induced by MINCLE appeared to be detrimental in a mouse model of pancreatic ductal adenocarcinoma. Authors demonstrated that ligation of MINCLE by its ligand SAP130, both highly expressed by inflammatory cells from tumors, promotes adaptive immune suppression and drives necrosome-induced accelerated oncogenesis (102). Interestingly, MINCLE not only acts as an activating (ITAM) receptor but can also act as an inhibitory-ITAM (ITAMi) receptor. Indeed, following recognition of the pathogen moieties of *Leishmania*, MINCLE shifts to an ITAMi configuration that impairs DC activation. Thus, ITAMi configuration exploited by a pathogen for immune evasion, may represent a paradigm for ITAM-coupled receptors sensing self and non-self (103, 104). As DAMPs are “mimics” of PAMPs, it will be interesting to investigate whether such a ligand-dependent dual sensing pathway exists also for a DAMP counterpart.

## CLEC12A (MICL, DCAL2, CLL-1, or CD371)

CLEC12A, is a homodimer expressed mostly by myeloid cells such as neutrophils, monocytes, macrophages and DCs, and is considered as a marker of acute myeloid leukemia blasts (22, 105–111). CLEC12A expression is downregulated by inflammatory stimuli (108, 112) and its ITIM domain recruits the tyrosine phosphatases SHP-1 and SHP-2 to counteract activating positive regulatory signals (22). CLEC12A was the first inhibitory receptor of the dectin cluster of genes described to sense a DAMP (Table 1 and Figure 1). It binds monosodium urate (MSU) crystal formed by crystallization of soluble uric acid following contact with extracellular sodium ions, when cells are dying. Thus, CLEC12A deficient mice, after *in vivo* challenge with MSU or necrotic cells or after radiation-induced thymocyte killing, exhibit hyperinflammatory responses (105). Functionally, CLEC12A limits the neutrophil recruitment in tissue following cell damage by inhibiting CXCL1 and CXCL10 production and by limiting ROS and IL8 production by neutrophils (105, 113). Therefore, by sensing cell death, CLEC12A represents an immune checkpoint that provides negative feedback mechanism for immunoregulation and protection of tissues from an overexuberant inflammatory response. As deposition of MSU crystal is observed in a variety of inflammatory responses such as in the arthritis disease “gout,” (105, 114), the development of a selective agonist for CLEC12A may represent a valuable therapeutic challenge. Interestingly, a link between CLEC12A and the type-I IFN response that is important for host immunity against viral infection, was recently demonstrated. Authors elegantly showed that following viral infection, CLEC12A triggered by MSU released by host dead cells, positively activates a type-I IFN response thereby amplifying anti-viral immune response (115). By sensing tissue integrity, CLEC12A can therefore finely modulate the equilibrium between infection-driven inflammation and control of pathogens. Authors proposed that as prolonged type-I IFN signaling during chronic virus infection facilitates virus persistence by inducing negative immune regulators, CLEC12A inhibition may be clinically beneficial in cases of persistent infection (116, 117). Similarly, the inhibitory receptor DCIR was shown to sustain type-I IFN signaling in DCs through interaction with an unidentified endogenous ligand(s) (118). Although both of these CLRs contain an ITIM in their cytoplasmic tail, and are thought to act as negative regulators of immune cell signaling, these results suggest that ITIM can somehow activate, rather than inhibit, some signaling pathways (22, 118). Whether these receptors deliver a signal on their own through the ITIM motif or require a co-receptor will need further molecular dissection. In fact, it will be interesting to investigate whether ITIM-coupled CLR-deficient animals develop autoimmune diseases as a consequence of impaired type-I IFN signaling, thereby increasing IL-12 production and Th1 differentiation (118). Interestingly, CLEC12A-deficient mice were reported to develop exacerbated arthritis in a collagen antibody-induced model characterized by cell death in the synovium (114). Astonishingly, authors proposed that during arthritis development, CLEC12A acts as an autoantigen that modulates threshold of myeloid cell activation. To support their hypothesis, they mentioned that this receptor is the target of autoantibodies in a subset of rheumatoid arthritis patients (114). Besides, several studies demonstrated in both mice and humans that CLEC12A on DCs serves as a specific target for antigen delivery to enhance CD8^+^ T cell and antibody responses (106, 110).

## CLEC9A (CD370, DNGR-1, UNQ9341)

CLEC9A is a homodimer highly expressed on common dendritic cell (DC) progenitors (CDPs) and type 1 conventional DCs (cDC1) (XCR1^+^ in mouse and BDCA3^+^ counterparts in human) (119–124). CLEC9A, whose expression is lost after Toll-like receptor-mediated maturation (122), binds to a fibrous polymer of actin, termed F-actin, an evolutionarily conserved ligand from yeast to mammals exposed by pathogens and dead cells (125, 126) (Table 1 and Figure 1). CLEC9A has a hemi-ITAM cytoplasmic tail with a highly conserved tyrosine that upon phosphorylation allows binding to SYK (23). Following F-actin recognition, CLEC9A signaling does not influence cell debris uptake nor maturation of dendritic cells (127). However, CLEC9A diverts phagocytosed dead cell cargo to a non-degradative recycling endosome compartment thereby facilitating cross-presentation of the dead-cell-associated antigens to CD8^+^ T cells (23). Myosin II, an actin-associated motor protein, potentiates the binding of CLEC9A to F-actin by facilitating co-operative binding of the two CTLD domains of the CLEC9A dimer, thereby rendering the cross-presentation more efficient (128). Thus, CLEC9A plays an important function in CD8^+^ T cell cross-priming during herpes virus infection (127, 129) and specifically induces optimal generation of tissue-resident memory T cells during influenza infection (130). The targeting of CLEC9A with tumor-expressed peptides together with adjuvant was shown to induce efficient cross-priming of CD8^+^ T cells and to control tumor immunity in a mouse model of melanoma (124). Moreover, during atherosclerosis, CLEC9A on cDC1, activated by the accumulation of necrotic cell debris, leads to disease progression by inducing macrophage and T-cell infiltration within lesions and by decreasing the expression of the anti-inflammatory cytokines TGFβ and IL-10 (131). Interestingly, in addition to its role in cross-presentation, CLEC9A exerts also an inhibitory feedback mechanism following tissue damage by activating the negative regulatory signal SHP-1 to dampen neutrophil-mediated immunopathology (132) (Figure 1). In this elegant study, authors showed that the lack of CLEC9A specifically in cDC1 increases the production of MIP-2 and consequently amplifies the recruitment of neutrophils and collateral tissue damage in mouse models of sterile and infectious injury (132). However, the mechanism by which F-actin could, according to the context, trigger opposing signals through SYK or SHP-1 remains to be elucidated. Taken together, these results suggest that the targeting of CLEC9A to regulate the antigenicity of dead or virus-infected cells, could have a clinical therapeutic impact for vaccination, infection and sterile inflammation.

## Conclusion

To conclude, we discussed in this mini review the recent studies that widened the array of identified responses elicited by these receptors and that shed light on the physiological and pathological functions of CLR in response to cell death. By sensing cell death, CLRs seem to protect against potential danger to cellular stress and excessive or deregulated cell death caused by non-infectious or infectious insults (133, 134). CLRs can therefore be considered as new immune checkpoint pathways acting as a safeguard to regulate the powerful and potentially harmful immune reactions and to prevent the accidental triggering of responses against the host's own tissues. As dysregulation of these checkpoint pathways induces development of diverse pathologies such as auto-immune diseases or cancers (40–42), understanding the mechanisms by which CLRs are triggered, beyond cell death, can pave the way for future targeting therapies (42, 134–136).

## Author Contributions

All authors listed have made a substantial, direct and intellectual contribution to the work, and approved it for publication.

### Conflict of Interest

MD was employed by the company OSE Immunotherapeutics. The remaining authors declare that the research was conducted in the absence of any commercial or financial relationships that could be construed as a potential conflict of interest.
